# Physician Awareness of Knee and Hip Pain in the Context of Coronary Heart Disease Treatment

**DOI:** 10.1155/2014/494801

**Published:** 2014-02-03

**Authors:** Mathias Glehr, Anna Kaltenbach, Reinhold Glehr, Patrick Sadoghi, Andreas Leithner, Gerald Gruber, Joanna Szkandera, Sabine Ludt, Ines Vielgut, Reingard Glehr, Michel Wensing

**Affiliations:** ^1^Department of Orthopaedic Surgery, Medical University of Graz, Auenbruggerplatz 5, 8036 Graz, Austria; ^2^Medical University of Graz, Auenbruggerplatz 5, 8036 Graz, Austria; ^3^Division of Clinical Oncology, Department of Internal Medicine, Medical University of Graz, Auenbruggerplatz 5, 8036 Graz, Austria; ^4^Department of General Practice and Health Services Research, Voßstraße 2, Geb. 37, 69115 Heidelberg, Germany; ^5^Department of General Practice and Family Medicine, Medical University of Vienna, Kinderspitalgasse 15/1, 1090 Vienna, Austria; ^6^Scientific Institute for Quality of Healthcare, Radboud University Nijmegen Medical Centre, P.O. Box 9101, 6500 HB Nijmegen, The Netherlands

## Abstract

*Background*. The benefit of physical activity for the prevention and treatment of cardiovascular disease (CVD) has been well documented. The aim of the present study was to determine the level of awareness among general practitioners (GPs) of knee and hip problems in patients with CVD or CVD risk. *Design*. Cross-sectional questionnaire survey. *Setting and Subjects*. Thirty-five Austrian GPs and 1,118 patients were included. The GPs completed an extraction form about the presence or absence of documented evidence of problems related to the knee and/or hip joint within the patient medical data. Patients, in turn, were asked to complete a questionnaire that included the Oxford Knee/Hip Score and the cardiovascular risk-chart established by the European Society of Cardiology. *Results*. In 748 patients' data from medical records and questionnaires were available. 40.9% of these patients suffered from serious knee pain and 32.1% from hip pain. However, in the medical records, in only 51.3% (knee) and 48.1% (hip) of these pain-patients the problems were documented. *Conclusion*. Joint disorders of the knee and hip problems are considerable barriers to effective physical activity and can therefore contribute to the development of CVD. Our data showed that GP awareness of such knee/hip disorders should be improved.

## 1. Background and Objective

The risk charts from the European Guidelines on Cardiovascular Disease Prevention focus on blood pressure, lipid profile, and smoking [1]. Regular physical activity and aerobic exercise training are recommended as a very important nonpharmacological treatment for primary and secondary CVD prevention [2–6]. However, to date, these guidelines have made no mention of the fact that osteoarthrotic pain in the knee and hip joint is a barrier to physical activity and should therefore be addressed during clinical consultation of CVD patients [1]. This is particularly problematic when one considers that patients with osteoarthritis seem to have higher CVD risk factors than the general population in all observed categories (hypertension, diabetes, high total cholesterol, low HDL cholesterol, and renal impairment or failure) [7].

Physical activity (PA) has been shown to inhibit the emergence and progression of CVD. It has beneficial effects on the development of atherosclerosis and results in a significant reduction in all-cause mortality [5]. Even in the more disabled patients, small amounts of properly supervised physical activity help improve cardiovascular status, maintain an independent lifestyle, and counteract disease-related depression [8, 9]. However, most patients with CVD do not reach a sufficient level of PA because a necessary lifestyle change cannot be performed. Since many of these patients are elderly, pain in the knee or hip joint may present a barrier to the modification of behaviour or lifestyle [3]. Meta-analyses of clinical studies have confirmed the positive effect of PA in the rehabilitation and therapy for patients suffering from CVD, myocardial infarction, or stroke [2, 5], which led to recommendations on PA in the secondary prevention of CVD [4, 10].

The aim of the present study was to determine the level of awareness among GPs of knee and hip problems in patients with CVD or CVD risk.

## 2. Methods

Over 100 GPs were selected by the Austrian Society of General Medicine and asked if they want to take part on a scientific project. The study was part of an international research project on cardiovascular risk management in primary care [11–13]. The protocol for this prospective study was approved by the Ethics Committee of the Medical University of Graz, and a written informed consent was obtained from all participants included.

Two samples of patients were included: patients with a documented CVD (*n* = 377 patients) and patients with a documented high risk of cardiovascular events (*n* = 371 patients). The sample with established CVD included patients with documented myocardial infarction or angina pectoris, as well as patients who had already had a vascular surgery or intervention. The sample “patients at high risk of cardiovascular events” was defined by meeting 3 of the following 4 criteria in the GP's documentation records: hypertension, hypercholesterolemia, smoking, and men over 60 years of age. Exclusion criteria were diabetes mellitus, terminal illness, cognitive impairment, psychiatric illness, and language problems [11, 13].

The patients consulted the GPs for miscellaneous reasons. They were not invited by the GP. Anonymity was guaranteed by forwarding merely the identification number of the questionnaire and extraction form. Identification was enabled by assigning one identification number for each patient and one for each GP. The GPs completed an extraction form about the presence or absence of documented evidence of problems related to the knee and/or hip joint within the patient medical data. Each patient had at least one consultation at the GP in the week before the GP completed the extraction form.

The patient's questionnaire was filled up by the patient at the day of visiting the GP. It contained items about joint status, restricted to the knee and hip. Symptoms in these joints were measured by the Oxford Scores, which are available for both knee—the Oxford Knee Score (OKS)—and hip—the Oxford Hip Score (OHS) [14–16]. Oxford Scores range from 0 to 48, with 0 indicating the worst functional joint status and 48 indicating the best. A total OKS or OHS between 30 and 39 points indicates mild or moderate knee or hip arthritis, while scores under 30 points represent severe osteoarthritis (OA) [14–16]. When assessing the prevalence of joint disorders in the three different groups, knee and hip disorders were evaluated separately. Patients were asked if they had had knee or hip pain in the past four weeks. Those who approved this condition were asked to complete the 12 questions of the Oxford Knee/Hip Score.

### 2.1. Statistical Methods

Documentation of the GP (patient suffering from knee or hip pain) was correlated with the OKS and OHS in the patient's questionnaire. The median value was used to evaluate the OKS and OHS scores because the distribution was not normal and the scores were based on a questionnaire (ordinal scale) for which the median is a more representative measure. As a measure of variation, the interquartile range (IQR) was calculated. For further statistical analysis, either Spearman's rank correlations or the Kruskal-Wallis tests were performed. Linear regression analyses were used to identify relationships between one dependent variable and one or more independent (predictor) variables. Linear regressions were used due to the fact that this analysis is a robust statistic which is resistant to deviations from assumptions. A *P*  value <0.05 was considered to be statistically significant. Statistical analysis was performed with SPSS software version 19 (SPSS, Chicago, IL; 2011).

## 3. Results

Thirty-eight Austrian GPs participated in the study—data from 35 GPs could be used because a lack of data quality from 3 GPs. Those 35 GPs provided data from 748 patients (Table 1). Some of the patients did not fulfil the complete questionnaire; for example, they did not write down their age. These missing files are mentioned in Table 1, demographic data.

### 3.1. Documentation of Knee and Hip Pain

In the study, 306 patients (40.9% of the CVD and CVD risk group) indicated (in our dichotomous item) that they had had knee pain during the last four weeks (Table 2). In 157 of these patients (51.3%), the GP had documented in the medical record that these patients suffer from knee pain. For the hip joint, 240 patients (32.1% of the CVD and CVD risk group) indicated that they had pain during the last four weeks. In the GPs' medical files, hip pain was documented in 113 patients (47.1%). There were patients with both knee and hip pain. The data of these patients were used for both knee and hip analysis because the Oxford Knee and Hip Scores are specially designed for one joint and ask for specific impairment that comes along with this joint.

No associations were found between the documentation of joint-related problems and the GP's professional experience, gender, or location (rural or urban area with 100,000 inhabitants or more).

The OKS was 36 in CVD group, (IQR = 14) and 37 in risk group (IQR = 11). The median OHS was statistically significantly different in the two groups (CVD group 35 (IQR = 13) and risk group 37 (IQR = 10)). Women had a significantly lower OKS (*P* = 0.02). There was no gender difference regarding the OHS.

## 4. Discussion

The aim of this study was to determine the level of awareness among general practitioners of knee and hip problems in patients with CVD or CVD risk. The GP is the primary contact for arthritis patients and the main care provider for most patients [17]. In our study, the participating GPs were only aware of patients' pain related to knee or hip problems in roughly half of the cases.

Prior studies also deal with joint-related pain in the lower extremity as a risk for CVD or GP's awareness of pain [18–22]. Regarding awareness of pain in a publication of Peters et al. 45% of the patients reported about knee problems, 24% about hip problems, and 31% about both [23]. In a large population based cohort study of Nüesch et al. 28% of the responders reported about pain in the knee and hip joint. They concluded that people with osteoarthritis of the knee and hip have an excess all-cause mortality compared with the general population, particularly pronounced for death from cardiovascular causes [24]. Marzolini et al. reported that over half (56%) of the patients with coronary artery diseases had musculoskeletal problems with joint pain accounting for 64.4% of these. Despite a greater need for comprehensive risk factor management in patients with musculoskeletal conditions, fewer patients were referred to cardiac rehabilitation [25]. Philbin et al. found that patients with lower extremity osteoarthritis may be at advanced risk for the development of cardiovascular heart disease by virtue of their unfavourable risk factor profile [26].

The “European Guidelines on Cardiovascular Disease prevention in clinical practice” mention both regular physical activity and aerobic exercise training to reduce the risk of fatal and nonfatal coronary events in healthy individuals, subjects with coronary risk factors, and cardiac patients over a wide age range [1]. However, these guidelines fail to mention that, in some cases, the patient may be unable to perform regular physical activity and exercise training because of pain in the lower extremities. When caring for patients with chronic diseases, doctors should remain alert to other disorders and minimize the number of missed opportunities for treating them [27]. This should also be highlighted in the guidelines for CVD prevention.

## 5. Limitations 

This study has several limitations. It might be sometimes common for older people to normalise joint pain and not report to the GP [28]. This lack of information could not be evaluated in this study. Also the potential ceiling effect of the OKS and OHS may have affected the current study, since patients can have musculoskeletal limitations without OKS or OHS identifying these.

## 6. Conclusion

Joint disorders of the knee and hip are considerable barriers to effective physical activity and can be considered as an independent CVD risk factor. Yet our data showed that there is considerable room for improvement in the GP awareness of such joint disorders when treating CVD. The guidelines on CVD prevention should therefore mention pain in the knee and hip joint as a barrier to physical activity and the related need to address joint problems during treatment for CVD. Further studies are necessary to evaluate joint disorders as CVD risk factor.

## Figures and Tables

**Table 1:** Demographic data of the patients.

	CVD group	Risk group	Total
*N* total	377	371	748
Female	129 (34%)	138 (37%)	267 (36%)
Mean age (SD)	69.43 (10.88)	64.71 (10.20)	67.09 (10.54)
Mean BMI (SD)	27.42 (4.84)	28.24 (4.24)	27.83 (4.54)
Mean OKS (SD)	34.13 (9.66)	36.56 (8.21)	35.33 (8.94)
Mean OHS (SD)	33.36 (9.14)	35.73 (7.75)	34.54 (8.45)

Standard deviation (SD); body mass index (BMI).

**Table 2:** Documentation of knee and hip pain by the GP in patients with documented CVD or documented risk for cardiovascular events.

Group	Knee pain		Hip pain	
	Yes	No	Yes	No
CVD documented	167	210	126	251
CVD risk group	139	232	114	257
∑	306	442	240	508

Pain documented by the GP	157 (**51.3%**)		113 (**47.1%**)	

## References

[1] Perk J, de Backer G, Gohlke H (2012). European guidelines on cardiovascular disease prevention in clinical practice (version 2012). The Fifth Joint Task Force of the European Society of Cardiology and Other Societies on Cardiovascular Disease Prevention in Clinical Practice (constituted by representatives of nine societies and by invited experts). *European Heart Journal*.

[2] Brown DA, Jew KN, Sparagna GC, Musch TI, Moore RL (2003). Exercise training preserves coronary flow and reduces infarct size after ischemia-reperfusion in rat heart. *Journal of Applied Physiology*.

[3] Hendry M, Williams NH, Markland D, Wilkinson C, Maddison P (2006). Why should we exercise when our knees hurt? A qualitative study of primary care patients with osteoarthritis of the knee. *Family Practice*.

[4] Li J, Siegrist J (2012). Physical activity and risk of cardiovascular disease-a meta-analysis of prospective cohort studies. *International Journal of Environmental Research and Public Health*.

[5] Taylor RS, Brown A, Ebrahim S (2004). Exercise-based rehabilitation for patients with coronary heart disease: systematic review and meta-analysis of randomized controlled trials. *The American Journal of Medicine*.

[6] Danielsen KK, Svendsen M, Maehlum S, Sundgot-Borgen J (2013). Changes in body composition, cardiovascular disease risk factors, and eating behavior after an intensive lifestyle intervention with high volume of physical activity in severely obese subjects: a prospective clinical controlled trial. *Journal of Obesity*.

[7] Singh G, Miller JD, Lee FH, Pettitt D, Russell MW (2002). Prevalence of cardiovascular disease risk factors among US adults with self-reported osteoarthritis: data from the third National Health and Nutrition Examination Survey. *The American Journal of Managed Care*.

[8] McCurry SM, Von Korff M, Vitiello MV (2011). Frequency of comorbid insomnia, pain, and depression in older adults with osteoarthritis: predictors of enrollment in a randomized treatment trial. *Journal of Psychosomatic Research*.

[9] Riddle DL, Kong X, Fitzgerald GK (2011). Psychological health impact on 2-year changes in pain and function in persons with knee pain: data from the osteoarthritis initiative. *Osteoarthritis and Cartilage*.

[10] Jorgensen TK, Nordentoft M, Krogh J (2012). How do general practitioners in Denmark promote physical activity?. *Scandinavian Journal of Primary Health Care*.

[11] Wensing M, Ludt S, Campbell S, van Lieshout J, Volbracht E, Grol R (2009). European practice assessment of cardiovascular risk management (EPA cardio): protocol of an international observational study in primary care. *Implementation Science*.

[12] Ludt S, Campbell SM, van Lieshout J, Grol R, Szecsenyi J, Wensing M (2011). Development and pilot of an internationally standardized measure of cardiovascular risk management in European primary care. *BMC Health Services Research*.

[13] van Lieshout J, Grol R, Campbell S (2012). Cardiovascular risk management in patients with coronary heart disease in primary care: variation across countries and practices: an observational study based on quality indicators. *BMC Family Practice*.

[14] Dawson J, Fitzpatrick R, Carr A, Murray D (1996). Questionnaire on the perceptions of patients about total hip replacement. *Journal of Bone and Joint Surgery B*.

[15] Dawson J, Fitzpatrick R, Murray D, Carr A (1998). Questionnaire on the perceptions of patients about total knee replacement. *Journal of Bone and Joint Surgery B*.

[16] Murray DW, Fitzpatrick R, Rogers K (2007). The use of the Oxford hip and knee scores. *Journal of Bone and Joint Surgery B*.

[17] Rosemann T, Körner T, Wensing M (2005). Rationale, design and conduct of a comprehensive evaluation of a primary care based intervention to improve the quality of life of osteoarthritis patients. The PraxArt-project: a cluster randomized controlled trial [ISRCTN87252339]. *BMC Public Health*.

[18] Cramm JM, Nieboer AP (2012). Factorial validation of the patient assessment of chronic illness care (PACIC) and PACIC short version (PACIC-S) among cardiovascular disease patients in the Netherlands. *Health and Quality of Life Outcomes*.

[19] Sanders C, Donovan JL, Dieppe PA (2004). Unmet need for joint replacement: a qualitative investigation of barriers to treatment among individuals with severe pain and disability of the hip and knee. *Rheumatology*.

[20] Davis GC, Hiemenz ML, White TL (2002). Barriers to managing chronic pain of older adults with arthritis. *Journal of Nursing Scholarship*.

[21] Gignac MAM, Davis AM, Hawker G (2006). “What do you expect? You’re just getting older”: a comparison of perceived osteoarthritis-related and aging-related health experiences in middle- and older-age adults. *Arthritis Care and Research*.

[22] Ang DC, Thomas K, Kroenke K (2007). An exploratory study of primary care physician decision making regarding total joint arthroplasty. *Journal of General Internal Medicine*.

[23] Peters TJ, Sanders C, Dieppe P, Donovan J (2005). Factors associated with change in pain and disability over time: a community-based prospective observational study of hip and knee osteoarthritis. *British Journal of General Practice*.

[24] Nüesch E, Dieppe P, Reichenbach S, Williams S, Iff S, Jüni P (2011). All cause and disease specific mortality in patients with knee or hip osteoarthritis: population based cohort study. *British Medical Journal*.

[25] Marzolini S, Oh PI, Alter D, Stewart DE, Grace SL (2012). Musculoskeletal comorbidities in cardiac patients: prevalence, predictors, and health services utilization. *Archives of Physical Medicine and Rehabilitation*.

[26] Philbin EF, Ries MD, Groff GD, Sheesley KA, French TS, Pearson TA (1996). Osteoarthritis as a determinant of an adverse coronary heart disease risk profile. *Journal of Cardiovascular Risk*.

[27] Redelmeier DA, Tan SH, Booth GL (1998). The treatment of unrelated disorders in patients with chronic medical diseases. *The New England Journal of Medicine*.

[28] Grime J, Richardson JC, Ong BN (2010). Perceptions of joint pain and feeling well in older people who reported being healthy: a qualitative study. *British Journal of General Practice*.

